# Evaluation and validation of candidate endogenous control genes for real-time quantitative PCR studies of breast cancer

**DOI:** 10.1186/1471-2199-8-107

**Published:** 2007-11-27

**Authors:** Roisin E McNeill, Nicola Miller, Michael J Kerin

**Affiliations:** 1 Department of Surgery, Clinical Science Institute, National University of Ireland, Galway, Ireland

## Abstract

**Background:**

Real-time quantitative PCR (RQ-PCR) forms the basis of many breast cancer biomarker studies and novel prognostic assays, paving the way towards personalised cancer treatments. Normalisation of relative RQ-PCR data is required to control for non-biological variation introduced during sample preparation. Endogenous control (EC) genes, used in this context, should ideally be expressed constitutively and uniformly across treatments in all test samples. Despite widespread recognition that the accuracy of the normalised data is largely dependent on the reliability of the EC, there are no reports of the systematic validation of genes commonly used for this purpose in the analysis of gene expression by RQ-PCR in primary breast cancer tissues. The aim of this study was to identify the most suitable endogenous control genes for RQ-PCR analysis of primary breast tissue from a panel of eleven candidates in current use. Oestrogen receptor alpha (*ESR1*) was used a target gene to compare the effect of choice of EC on the estimate of gene quantity.

**Results:**

The expression and validity of candidate ECs (*GAPDH*, *TFRC*, *ABL*, *PPIA*, *HPRT1*, *RPLP0*, *B2M*, *GUSB*, *MRPL19*, *PUM1 *and *PSMC4) *was determined in 6 benign and 21 malignant primary breast cancer tissues. Gene expression data was analysed using two different statistical models. *MRPL19 *and *PPIA *were identified as the most stable and reliable EC genes, while *GUSB*, *RPLP0 *and *ABL *were least stable. There was a highly significant difference in variance between ECs. *ESR1 *expression was appreciably higher in malignant compared to benign tissues and there was a significant effect of EC on the magnitude of the error associated with the relative quantity of *ESR1*.

**Conclusion:**

We have validated two endogenous control genes, *MRPL19* and *PPIA*, for RQ-PCR analysis of gene expression in primary breast tissue. Of the genes in current use in this field, the above combination offers increased accuracy and resolution in the quantitation of gene expression data, facilitating the detection of smaller changes in gene expression than otherwise possible. The combination identified here is a good candidate for use as a two-gene endogenous control in a broad spectrum of future research and diagnostic applications in breast cancer.

## Background

Breast cancer is the most common form of malignancy among women in almost all of Europe and in North America. Each year over one million women worldwide are diagnosed with the disease and it causes over 400,000 deaths annually. Much of the current translational research in this area is based on "transcriptomics", the elucidation of the transcriptional programs underlying disease initiation, promotion and progression, through tumour gene expression profiling. Real-time quantitative PCR (RQ-PCR) [1,2] is one of the most sensitive and specific quantitation methods for gene expression analysis and is firmly established as a mainstream research tool [3-5]. With the development of high throughput and reliable instrumentation, improved detection chemistries, more efficient protocols and appropriate analysis software, RQ-PCR has become the basis of many breast cancer biomarker studies as well as several novel diagnostic and prognostic assays [6-12]. In addition, RQ-PCR is used to validate microarray expression profiles and quantify genes of interest identified from those analyses.

The most commonly used method to quantify gene expression involves the analysis of target gene expression relative to a control gene. As in other relative gene expression analysis techniques such as Northern blotting and ribonuclease protection assays (RPAs), normalisation of RQ-PCR data is required to control for variation introduced during the steps from RNA extraction to quantitation, especially to control for differences in the quantity and quality of RNA used in reactions [13]. The use of endogenous control (EC) genes, known variously as housekeepers, reference or simply control genes is based on the principle that these genes are expressed constitutively and uniformly in all test samples, so that expression of the target gene can be normalised against them to control for systematic variation in sample handling. Results are then expressed as the ratio of target gene expression relative to the EC gene. In many cases, control genes are inherited from earlier studies using less sensitive forerunner techniques such as Northern blotting and little if any consideration has been paid to validating these genes as controls for specific experiments.

Two of the most commonly used endogenous control genes for breast cancer gene expression studies are glyceraldehyde-3-phosphate dehydrogenase (*GAPDH*) and β-actin (*ACTB*) but their reliability in this context has not been demonstrated. In some studies the use of either gene may be inappropriate, as these genes have been implicated in aspects of disease aetiology [14-20]. For example, bisphosphonates; used to inhibit bone resorption in diseases including osteoporosis, Paget's disease and metastatic breast cancer, target *GAPDH*, decreasing its expression in both breast and prostate cell lines [21], while the actin filament protein family, of which *ACTB *is a member, may be modulated in malignancy [22] particularly during processes involving reorganisation of the cytoskeleton such as invasion and migration.

The precision of the estimate of change in target gene expression is dependent on the stability of the endogenous control, the variability associated with the target gene and any covariance between the two. Thus the use of non-validated endogenous control genes results in, at best, unreliable data. It is now recognised that a universal, invariably expressed gene is unlikely to exist [23] and may not exist even within individual tissue or cell types. The goal therefore is to identify the most reliable gene or set of genes as endogenous controls for a particular experiment. As a result of this, several groups have developed statistical models and software programs for the analysis of candidate gene stability. The aim of this study was to identify the most stable endogenous control genes from a panel of eleven candidates commonly used as endogenous controls in the context of, but not limited to, breast cancer: *GAPDH, TFRC, ABL, PPIA, HPRT1, RPLP0, B2M, GUSB, MRPL19, PUM1*, *PSMC4*, for the quantification of gene expression by relative comparative RQ-PCR in primary breast cancer tissues. The oestrogen receptor alpha (*ESR1*) transcript, a gene of special significance in breast cancer, was used as a target gene to compare the effect of choice of EC on the estimate of gene quantity.

## Results

To identify suitable EC genes for breast cancer gene expression studies in fresh-frozen primary tissue, a panel of 11 genes commonly used as ECs was selected from the literature for analysis of stability: *GAPDH, TFRC, ABL, PPIA, HPRT1, RPLP0, B2M, GUSB, MRPL19, PUM1 *and *PSMC4*. Genes were analysed in tumours recovered from patients with benign or malignant breast disease using RQ-PCR. Stability of candidate EC genes was analysed using two statistical analysis tools, geNorm and NormFinder, which employ different statistical models to define the most reliable EC genes for normalisation. The effect of choice of EC gene on target gene expression was analysed using *ESR1 *as target.

### Range of expression of candidate EC genes and *ESR1*

The candidate ECs displayed a range of C_t _values. Mean C_t _values per gene and the range of C_t _values for each gene are shown in Table 1. Mean C_t _values ranged from 19.13 (± 0.21 s.e.m.) for *B2M *to 26.48 (± 0.15 s.e.m.) for *MRPL19*.*MRPL19 *showed the narrowest range followed by *PPIA*. The genes broadly fell into two categories, those highly expressed with mean C_t _values of 19–20 (*B2M, RPLP0, GAPDH, PPIA*) and moderate abundance genes with mean C_t _values of 23–26 (*PSMC4, ABL, GUSB, TFRC, PUM1, HPRT1 *and *MRPL19*). The target gene *ESR1 *showed the broadest range of C_t _values (10.35) from 17.10–27.45.

**Table 1:** Cycle threshold (C_t_) values of candidate EC genes and *ESRI*. Among the candidate ECs, *MRPL19 *and *PPIA *showed the smallest range in C_t _values while *GAPDH*, *HPRT1*, and *RPLP0 *showed the greatest. Candidates fell into two groups in terms of abundance, high (C_t _19–20; *B2M, RPLP0, GAPDH *and *PPIA*) and moderate abundance (C_t _23–26; *PSMC4, ABL, GUSB, TFRC, PUM1, HPRT1 *and *MRPL19*). *ESR1 *ranged over 10.35 C_t _values

**Gene symbol**	**C_t _Range**	**C_t_Min**.	**C_t_Max**.	**Mean C_t _± s.e.m**.
*MRPL19*	4.06	24.79	28.84	26.48 ± 0.15
*PPIA*	4.4	18.01	22.41	19.74 ± 0.18
*B2M*	4.67	17.22	21.89	19.13 ± 0.21
*PUM1*	5.06	22.38	27.44	24.78 ± 0.21
*GUS*	5.19	21.37	26.55	23.94 ± 0.20
*PSMC4*	5.69	20.84	26.52	23.32 ± 0.22
*ABL*	5.89	21.27	27.15	23.75 ± 0.30
*TFRC*	6.03	21.4	27.42	24.40 ± 0.29
*GAPDH*	6.88	16.79	23.67	19.71 ± 0.25
*HPRT1*	7.05	21.57	28.61	24.84 ± 0.27
*RPLPO*	8.8	16.1	24.9	19.46 ± 0.33
*ESR1*	10.35	17.10	27.45	21.96 ± 0.34

**Figure 1:**
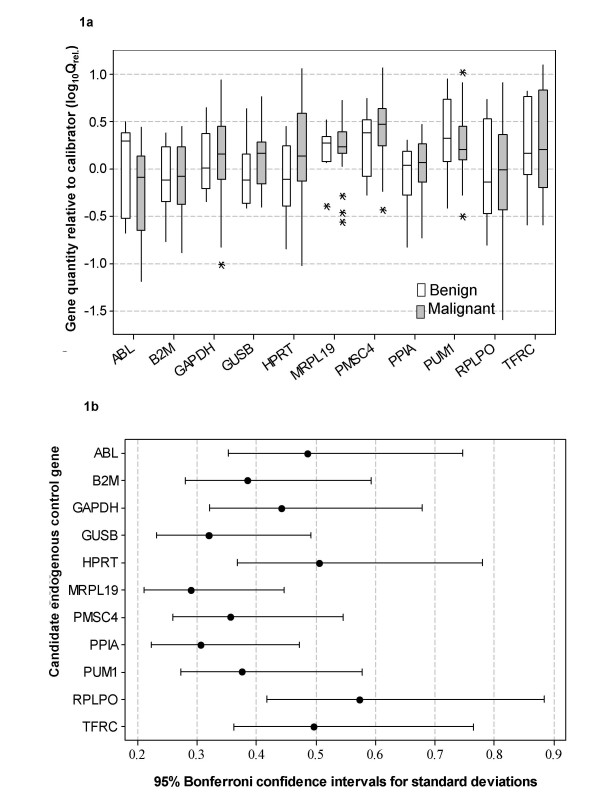
**Relative quantity and variation associated with each candidate EC**. a) Quantity of candidate endogenous control genes *GAPDH, TFRC, ABL, PPIA, HPRT1, RPLP0, B2M, GUSB, MRPL19, PUM1 *and *PSMC4 *in benign and malignant breast tumour tissue relative to calibrator (Q_Rel_. = E^-ΔCt^). Boxplot shows median value, interquartile-range box and outliers (*). Within gene there was no difference in gene quantities between benign and malignant tissues (*P *> 0.05). b) Variation associated with candidate endogenous control genes *GAPDH, TFRC, ABL, PPIA, HPRT1, RPLP0, B2M, GUSB, MRPL19, PUM1 *and *PSMC4 *in all breast tumours relative to calibrator. Relative gene expression was calculated using the ΔC_t _method and corrected for efficiency of amplification (Q_Rel_. = E^-ΔCt^). There was a significant difference in variance associated with relative gene expression (*P *= 0.001) with genes such as *RPLP0*, *TRFC*, *HPRT1 *and *GAPDH *showing greater variance than genes such as *MRPL19 *and *PPIA*.

With conversion of C_t _values to relative quantity values (Q_Rel_.), there was no difference in candidate EC gene quantities between benign and malignant tissues (*P *> 0.05; Fig. 1a). There was however, a significant difference in variance between genes (*P *= 0.001; Fig. 1b) indicative of differing stabilities of the candidates.

### Analysis of EC gene stability

The significant difference in EC variability demonstrated the necessity to validate their use in this context. Expression stability was analysed using the two softwares geNorm [24,25] and NormFinder [26].

**Figure 2:**
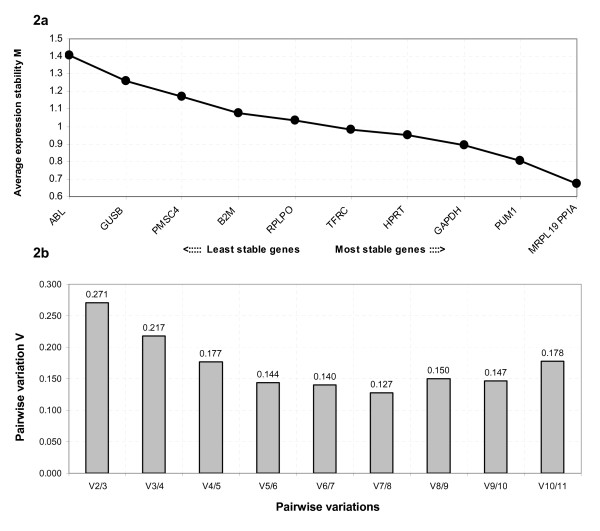
**GeNorm analysis of the candidate EC genes**. Results are presented as per the output file of the geNorm programme [24]. (a) Stepwise exclusion of the least stable genes. The gene stability value *M *is based on the average pairwise variation between all tested genes. Low *M *values characterise genes with greater stability, thus the x-axis from left to right indicates the ranking of the EC genes according to expression stability and the y-axis indicates the stability measure, *M*. (b) Determination of the optimal number of ECs for normalisation. The recommended upper limit of the pairwise variation value *V *is set at 0.15 but in meeting this criterion, sample availability, the practicality of using multiple EC genes and the degree of required resolution must be considered.

GeNorm uses a pair-wise comparison-based model to select from a panel of candidate EC genes, the gene-pair showing least variation in expression ratio across samples. It calculates a measure of gene stability (*M*) of each gene based on the average pairwise variation between all tested genes. Genes with the lowest *M *values are those demonstrating most stable expression. Table 2 shows the *M *values for all tested genes. Eight of the eleven genes analysed; *PPIA*, *MRPL19*, *GAPDH*, *PUM1*, *B2M*, *HPRT1*, *PSMC4 *and *TFRC*, showed *M *values less than the geNorm default threshold of 1.5, while the three remaining genes; *RPLP0*, *GUSB *and *ABL*, showed *M *values greater than that threshold. In a stepwise progression, geNorm excludes the least stable gene, recalculating *M *for the remaining genes, resulting in the characterisation of the stability of each gene on a ranked scale and ultimately the identification of the two most stably expressed genes (Fig. 2a). As shown,*ABL *and *GUSB *were the first and second genes respectively excluded from the analysis on the basis of instability and *MRPL19 *and *PPIA *were identified as most stable gene-pair.

**Table 2:** Expression stability values of EC genes calculated by geNorm and NormFinder programmes

	**NormFinder**		**geNorm**	
**Rank**	**Gene**	**Stability^b^**	**Gene**	**Stability**
1	*MRPL19*	0.105	*PPIA*	1.116
2	*PPIA*	0.119	*MRPL19*	1.134
3	*B2M*	0.169	*GAPDH*	1.240
4	*GAPDH*	0.176	*PUM1*	1.275
5	*PUM1*	0.222	*B2M*	1.305
6	*HPRT1*	0.233	*HPRT1*	1.357
7	*TFRC*	0.236	*PSMC4*	1.419
8	*PSMC4*	0.247	*TFRC*	1.437
9	*GUSB*	0.268	*RPLPO*	1.549
10	*RPLPO*	0.295	*GUSB*	1.568
11	*ABL*	0.413	*ABL*	2.070

^b^High expression stability is indicated by a low stability value. For geNorm, stability is based on an estimate of the pairwise variation (*M*) relative expression while for NormFinder, stability is based on combined inter- and intra-group variation.

GeNorm also calculates a normalisation factor (NF) required to determine the optimal number of EC genes required for accurate normalisation. This factor is calculated using the variable *V *as the pairwise variation (V_n_/V_n + 1_) between two sequential NFs (NF_n _and NF_n + 1_). To meet the recommended cut off *V*-value of 0.15, the point at which it is unnecessary to include additional genes in a normalisation strategy [24], the programme indicated the use of 5 of the six most stable genes *i.e*., *MRPL19*, *PPIA*, *GAPDH*, *PUM1 *and *B2M *(Fig. 2b). However, there was no significant effect on relative quantity of *ESR1 *expression using the 5 gene panel of *MRPL19*, *PPIA*, *B2M GAPDH *and *PUM1*, compared to the two-gene combination of *MRPL19 *and *PPIA *(*P *> 0.05).

Stability of gene expression was also analysed using NormFinder [26]. This programme uses a combined estimate of the intra- and inter-group variation to determine the most stably expressed candidate EC gene and gene-pair. Table 2 shows the ranking of the candidates. As for geNorm, NormFinder identified *MRPL19 *and *PPIA *as the most stable pair of genes and *MRLP19 *as the single most stable gene.

### Associations between candidates EC genes and *ESR1*

The geNorm programme assumes no co-regulation of candidate ECs as obviously this would lead to an erroneous choice of optimum normaliser pair. As stated above, to our knowledge the candidate ECs tested in this study are functionally independent. In addition co-variance between target gene and EC would affect results. Regression analysis demonstrated significant, negative, linear associations between the relative quantities (Q_Rel_.) of the target gene, *ESR1 *and two commonly used ECs; *TFRC *(*TFRC *= 0.079 - 0.272 *ESR1*; R^2 ^= 0.18; *P *< 0.05) and *HPRT1 *(*HPRT1 *= - 0.202 - 0.386 *ESR1*; R^2 ^= 0.35; *P *= 0.001). In addition there was a significant linear association between Q_Rel_. of *ESR1 *and *ABL *described by the equation: *ABL *= 0.044 + 0.290 *ESR1*; R^2 ^= 0.21;*P *= 0.012. There was no association between *ESR1 *and either *MRPL19 *or *PPIA *(*P *> 0.05).

### Effect of EC on *ESR1 *relative gene expression

**Figure 3:**
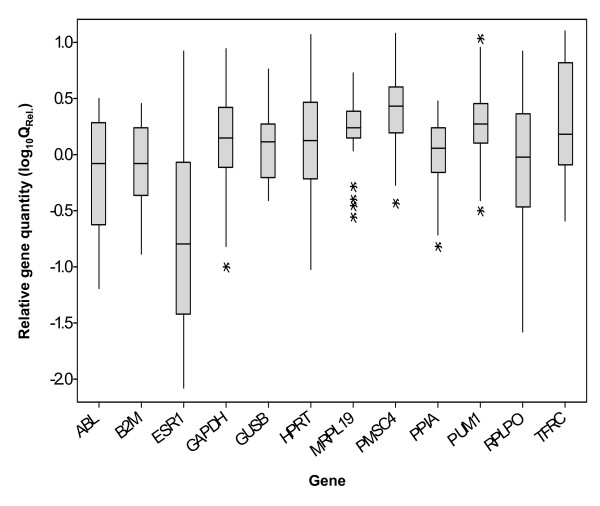
**Quantity of all genes expressed in breast tissues relative to calibrator (Q_Rel_. = E^-ΔCt^)**. A pool of cDNA from two normal tissues was used as calibrator.

**Figure 4:**
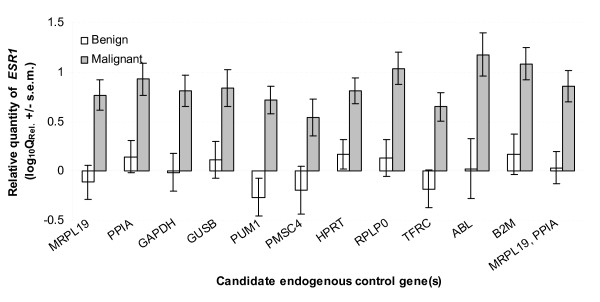
**Relative quantity of oestrogen receptor alpha mRNA (*ESR1*) in benign and malignant breast tumour tissue**. Quantity of gene expression was calculated relative to each candidate endogenous control gene and to the geometric mean of *MRPL19 *and *PPIA *(Q_Rel_. = E^-ΔΔCt ^± s.e.m.). A pool of cDNA from two normal tissues was used as calibrator. There was no effect of EC on the relative quantity of *ESR1 *in either group (*P *> 0.05), however, there was a significant effect of EC gene on the estimate of the error associated with relative gene expression (*P *< 0.05). The error was significantly reduced using the combination of *MRL19 *and *PPIA *compared to the use of all EC genes individually with the exception of *MRPL19*.

There was no effect of choice of EC on the relative quantity of *ESR1 *(*P *> 0.05) probably due to the large variation associated with the expression of *ESR1 *compared to any of the EC genes as shown in Fig. 3. However, there was a significant effect of EC gene on the estimate of the error associated with relative gene expression (*P *< 0.05). The magnitude of the error was significantly reduced using *MRL19 *and *PPIA *as a combined endogenous control compared to the use of *ABL*, *B2M*, *GAPDH*, *GUSB*, *HPRT1*, *PSMC4*, *PPIA*, *PUM1*, *RPLP0 *and *TFRC*. While there was no difference in error using *MRPL19 *and *PPIA *in combination compared to *MRPL19 *on its own (*P *> 0.05), there was a trend towards a reduction in the estimate of the error using the combined normalising factor. There was a significant increase in relative quantity of *ESR1 *in malignant versus benign breast tissues compared to normal breast tissue (*P *< 0.01; Fig. 4).

## Discussion

To our knowledge this is the first systematic evaluation of the reliability of a large number of genes used as endogenous controls for RQ-PCR analysis in breast cancer studies. The literature cites just two articles in relation to the evaluation of EC genes for breast cancer [27,28]. However, one study characterised ECs not in primary breast tissues but in cell lines [27] and the other, while employing primary breast tissues, compared just two commonly used EC genes with other genes selected from a microarray dataset of breast cancer tissues and cell lines, as well as cell lines of different origins [28].

Quantitative PCR is the basis of most nucleic acid-based breast cancer biomarker studies and its potential clinical utility is foretold by the development of the Onco*type* Dx assay (Genomic Health). This 21-gene assay can predict metastatic recurrence [11] and magnitude of response to chemotherapy [29] in Tamoxifen-treated ER-positive early breast cancer patients. RQ-PCR will undoubtedly feature prominently in the move toward personalised medicine so the necessity of validating ECs in clinical samples as opposed to cell lines is clear. The diversity of the tissues used in this study in terms of histological and clinical parameters (Table 3) makes the results of interest to a broad spectrum of the breast cancer research community. With the exception of *ABL*, used as an EC in other settings [30], genes were selected for evaluation based their prior use in breast cancer studies, to determine the most reliable EC of those used in this field. Certain genes were excluded based on evidence that their use in this context is inappropriate [20,22,31-33].

**Table 3:** Clinical and histological data relating to the benign (Ben.) and malignant (Mal.) breast tissues. Data includes patient menopausal status and histological type, and tumour size, T, N, M, UICC stage, grade, ER, PR and HER2/*neu *status and intrinsic subtype of malignant tissues where available

**Tissue type**	**Size (mm)**	**T**	**N**	**M**	**UICC**	**Grade**	**Menopausal status**	**Histological type**	**Subtype**	**ER**	**PR**	**HER2/*neu***
Mal.	35	2	1	0	2B	3	pre	ductal				
Mal.	22	2	1	0	2B	2	post	ductal	luminal A	8	4	negative
Mal.	22	2	0	0	2B	3	pre	ductal	basal	0	0	negative
Mal.	25	2	0	0	2A		pre	ductal	unknown			
Mal.	37	2	1	0	2B	3	pre	ductal	luminal A	8	8	negative
Mal.	35	2	1	0	2B	1	pre	lobular	luminal A	7	8	negative
Mal.	45	2	1	0	2B	3	pre	ductal	basal	0	0	negative
Ben.							pre	fibrocystic				
Mal.	20	1	1	0	2A	1	post	ductal	luminal A	8	8	negative
Mal.	50	2	1	0	2B	3	post	ductal	luminal B	8	3	positive
Mal.	15	1	1	0	2A	2	post	ductal	luminal A	8	3	negative
Mal.	20	1	0	0	1	2	post	ductal	luminal A	8	8	negative
Mal.	25	4	1	0	3B	3	post	ductal	luminal B	4	4	positive
Mal.	10	1	0	0	1		post	ductal, some tubular	luminal A	8	8	negative
Mal.	33	2	1	0	2B	1	post	lobular	luminal A	8	8	negative
Mal.	30	2	1	0	2B	3	pre	ductal	luminal A	7	8	negative
Mal.	30	2	1	0	2B	3	pre	ductal		0	0	
Mal.	20	1				1	post	colloid/mucinous				
Mal.	40	2	1	0	2B	2	post	lobular	luminal B	8	0	positive
Ben.												
Ben.							pre	fibroadenoma				
Ben.							pre	parenchymal inflammation				
Mal.	35	4	2	0	3B	3	post	ductal	luminal A	8	8	negative
Mal.	35	2	1	0	2B	3	post	ductal	luminal A	8	6	negative
Mal.							pre	ductal	luminal A	7	0	negative
Mal.	25	2	0	0	2A	2	pre	ductal	Her2	0	0	positive
Mal.	60	4	2	1	4	2						
Ben.							pre					
Ben.							pre	fibroadenoma				

Abbreviations: T: size or extent of primary tumour; N: spread to regional lymph nodes; M: distant metastasis; UICC, tumour stage according to the International Union Against Cancer TNM classification; ER: oestrogen receptor status; PR: progesterone receptor status; HER2/*neu*: v-erb-b2 erythroblastic leukaemia viral oncogene status.

Validation of EC genes raises the circular issue of how to normalise normalising genes. This issue governs the validity of the conclusions of such studies so at each stage of this experiment sources of non-biological variation were minimised and data were scaled relative to a calibrator. For example, RNA integrity, quality and purity were stringently analysed. A threshold RIN value of 7 was applied, below which samples were excluded from analysis. This aspect is of importance given the relationship between RNA integrity and expression quantitation [34-36]. Duplicate cDNA reactions were performed and genes were amplified in triplicate using more stringent cut-offs for replicate variability than recommended elsewhere [37]. In addition, the efficiency of amplification of each assay was determined (Table 4) and data were corrected appropriately. Determination of assay efficiency is critical in comparing gene expression [38] but has not been addressed in similar studies [39]. Cycle threshold (C_t_) data were scaled relative a pooled normal tissue calibrator. Similar studies describe the comparison of genes based on raw C_t _values [40,41], an inappropriate approach as discussed below and elsewhere [36].

**Table 4:** Details of gene-specific RQ-PCR assays

**Gene symbol**	**Gene name**	**Molecular function**	**Applied Biosystems assay identifier**	**Amplicon size (bp)**	**Slope of inhibition curve**	**PCR Amplification efficiency (%)**
*ABL*	Abelson murine leukaemia viral 1	non-receptor tyrosine protein kinase	Hs00245443_m1	54	-3.47	93.9
*B2M*	Beta-2-microglobulin	defense/immunity protein	4333766	75	-3.48	93.6
*GAPDH*	Glyceraldehyde-3-phosphate dehydrogenase	oxidoreductase, dehydrogenase	4333764	168	-3.52	92.3
*GUSB*	Glucuronidase, beta	galactosidase	4333767	63	-3.38	97.3
*HPRT1*	Hypoxanthine guanine phosphoribosyl transferase 1	glycosyltransferase	4333768	100	-3.37	97.7
*MRPL19*	Mitochondrial ribosomal protein L19	protein biosynthesis	Hs00608519_m1	72	-3.14	107.7
*PPIA*	Peptidylprolyl isomerase A	isomerase	Hs99999904_m1	98	-3.38	97.3
*PSMC4*	Proteasome 26S subunit, ATPase, 4	protease, hydrolase	Hs00197826_m1	83	-3.38	97.6
*PUM1*	Pumilio, Drosophila, homolog of, 1	RNA binding, translation regulation	Hs00982776_m1	62	-3.30	100.7
*RPLP0*	Ribosomal protein, large, P0	protein biosynthesis	4333761	154	-3.51	92.7
*TFRC*	Transferrin receptor	ion receptor	4333770	130	-3.56	90.9
*ESR1*	Oestrogen receptor alpha	nuclear steroid receptor	Hs00174860_m1	62	-3.45	94.5

There was no effect of tissue type on EC expression, validating comparison of their stability. This is an essential but often overlooked precursor analysis when using geNorm and NormFinder [42] since these methodologies assume the candidates are not differentially expressed between experimental groups. There was however a significant difference in variance between candidates (*P *= 0.001; Fig. 1), with genes such as *RPLP0*, *TRFC*, *HPRT1 *and *GAPDH *showing greater variance than others *e*.*g*., *MRPL19 *and *PPIA*. Since the resolution of RQ-PCR is defined by the variance associated with the EC [13] these results emphasise the necessity to evaluate and validate EC genes.

A single universal EC is unlikely to exist [43] and since the function of most genes is largely unknown it is impossible to predict their expression under different experimental conditions. The use of more than one EC hedges the bet and increases the accuracy of quantitation compared to the use of a single EC [13,24,26,36,44]. Studies show substantial errors, up to 6.5-fold, in expression quantitation using single as opposed to multiple EC genes [24]. In this study, stability of expression was analysed using two distinct statistical models, a pairwise comparison model, geNorm, and an ANOVA-based model, NormFinder. The geNorm applet selects from a panel of genes, the pair showing least variation in expression ratio across samples and estimates the minimum number of genes required for optimal normalisation. NormFinder estimates stability values for ECs considering combined intra- and inter-group variation and identifies the most stable gene and gene-pair, where the stability of the pair exceeds that of the single gene. Despite their differences both models identified *MRPL19 *and *PPIA *as the most reliable ECs while *RPLP0*, *GUSB *and *ABL *were least reliable. This result reflects those of the equality of variance analysis and, broadly, ranking by range of C_t _values (Table 2). However, the ranking of genes by C_t _range and by the model-based methods differed for some genes *e*.*g*., *GAPDH*, demonstrating the necessity to scale and correct raw C_t_s for amplification efficiency before analysis. GeNorm indicated that optimal normalisation could be achieved using the five most stable genes but there was no difference in *ESR1 *gene expression using this approach as opposed to the two-gene combination. While it is not known whether this would hold for other less variable target genes, cost and sample availability are limiting factors for most studies so the two-gene combination may be more practical for most applications.

The effect of using less stable ECs was assessed using *ESR1 *as a target gene. Due to the high variability of the expression of this gene (Fig. 4) there was no effect of EC on quantitation. Without further discussion of the relevance of the differential expression of *ESR1 *in benign and malignant tissues; a gene whose role in breast cancer is widely appreciated, it is likely that had the target gene shown a more discreet change in gene expression, an effect of EC on quantitation would have been apparent. There was however a clear tendency for *ESR1 *expression to change depending on EC (Fig. 4). In the benign samples *ESR1 *could be made to appear up- or down-regulated depending on EC, while its expression in the malignant samples could be numerically altered by one order of magnitude – artifactual results due simply to the choice of EC. Clearly this type of error is unacceptable, especially in the analysis of markers for potential clinical application.

Furthermore, there was a significant effect of EC on the magnitude of the error associated with the estimate of *ESR1 *expression. The use of the *MRPL19*, *PPIA *combination minimised the error compared to all other ECs with the exception of *MRPL19*. Apart from the fact that the use of single EC genes can compromise data as already described, the use of either gene alone is not recommended for two further reasons. Firstly, geNorm identified them based on a pairwise comparison model so their individual use is inconsistent with that analysis method. As shown in Fig. 4, *ESR1 *expression can be made numerically increase or decrease in the benign groups if these genes are used singly. Secondly, stability, as assessed by NormFinder, increased roughly 30 percentage points, from 0.105 using *MRPL19 *alone to 0.072 using it in combination with *PPIA *indicating improved reliability of the two-gene combination [26].

One previous study analysed EC stability in primary breast tissue [28]. That study compared two traditionally used ECs, *GAPDH *and *ACTB *and four genes identified in microarray studies [45,46]: *MRPL19*, *PUM1*, *PSMC4 *and *SF3A1*. The authors recommended *MRPL19 *be used with *PSMC4 *and *PUM1*. However, in the present study, *PPIA*, which was not assessed by Szabo and colleagues, showed greater stability than *PSMC4 *and *PUM1*. Of the genes analysed in this study, *PPIA *ranked first and second by Normfinder and geNorm respectively compared to fifth and seventh for *PSMC4 *and eighth and fourth for *PUM1*. The reason(s) *PPIA *did mot emerge as a candidate in Szabo and colleagues' microarray study is unclear. As part of their selection procedure, data was filtered to remove near background signals from low abundance genes yet in this study *PPIA *showed the third highest mean C_t _value. Cohort-specific effects are also unlikely since the tissues are broadly similar in terms of their clinical and histological parameters. Apart from the obvious differences in the quantitative capacities of microarray and RQ-PCR technology, a possible explanation is that the cohort from which these authors selected their candidates was not breast cancer-specific and included tissue from metastatic breast cancer lymph nodes and cell lines from an assortment of origins including dermal, leukemic, umbilical and melanoma samples [46].

This study also analysed associations between genes. An assumption of the geNorm model is that candidate ECs are not co-regulated yet the analysis of such genes by that method would lead to an erroneous choice of best gene-pair. To our knowledge the EC genes evaluated here are functionally independent as shown in Table 4. In addition, covariance of target and EC is clearly unacceptable. However, regression analysis showed significant linear relationships between *ESR1 *and three of the candidates: *TFRC*, *HPRT1 *and *ABL*. Approximately two thirds of breast tumours are oestrogen-dependent and the number of genes whose expression is known to be, or likely to be mediated through the receptor is sizeable [47]. This makes analysis of associations between oestrogen-responsive target and control genes of particular importance in breast cancer studies. Although there is little evidence that these genes are regulated by oestrogen, the results suggest that aside from their poor stability it would be wise not to use them as ECs in the analysis of oestrogen-responsive breast cancer.

Despite the clear increase in accuracy afforded by the use of more than one validated EC gene, a recent survey of working practices indicated that over half of those polled continue to use one reference gene and that two thirds of these do not validate that gene [48]. Obviously levels of awareness and/or willingness to address this issue must be improved.

## Conclusion

The current emphasis on personalised cancer treatment has resulted in the development of prognostic and predictive multi-gene RQ-PCR assays. However, with such developments comes the demand for greater accuracy and resolution of gene expression quantitation. In this study we have validated two genes, *MRPL19 *and *PPIA *as EC candidates for RQ-PCR analysis of primary breast tissue using two different statistical models and demonstrate that of the genes in current use in this field, the above gene combination offers increased accuracy and resolution in the relative quantitation of gene expression data. The genes identified should be of use in a broad spectrum of translational research and diagnostic applications in breast cancer.

## Methods

### Breast tissue samples

Primary breast tumour tissues (n = 27) were obtained from patients during primary curative resection, at Galway University Hospital, Galway, Ireland. Samples were categorised into benign (n = 6) or malignant groups (n = 21) according to analysis of standard histopathological parameters. Clinical data relating to the tumour tissues used in this study are shown in Table 3. RNA from normal tissues, recovered from patients undergoing reduction mastopexy were used as calibrator samples for comparative relative RQ-PCR (n = 2). After excision, tissue samples were immediately snap-frozen in liquid nitrogen and stored at -80°C until RNA extraction. Prior written and informed consent was obtained from each patient and the study was approved by the ethics review board of Galway University Hospital. Clinical data were obtained from the Breast Cancer Database at the Department of Surgery, Galway University Hospital.

### Candidate endogenous control genes

Eleven commonly used candidate endogenous control genes were selected for analysis (Table 4). To our knowledge, all genes are constitutively expressed in breast cancer tissues and all have independent cellular functions and are assumed not to be co-regulated. Only *RPLP0 *and *MRPL19 *share a molecular function, *i.e*., protein biosynthesis.

### Minimisation of non-biological variation

While target gene expression is normalised using EC genes to correct for variation introduced during sample processing using, this is obviously not possible in EC validation studies. Since this critical issue governs the reliability of the data generated and the validity of the conclusions it was addressed as described below.

Firstly, while it was not possible to control for variation in the acquisition of clinical samples collected over a number of years, every effort was made to minimise systematic variation downstream of sample acquisition. All equipment and instruments were calibrated before use. Benign and malignant samples were homogenised separately but on the same day. All RNA was extracted using the same protocol and reagent lot by one person to avoid batch-to-batch variation. Where possible, two extractions from separate areas of the each tissue sample were pooled to control for tissue heterogeneity. RNA integrity and purity were stringently analysed as described below. Duplicate cDNA reactions were performed to minimise variation from the reverse transcription step. No-RT controls were included with each batch of cDNA synthesised. All PCR reactions were performed on the same pool of aliquotted cDNA and no-template controls were included in each run for each gene. Appropriate inter-assay controls were included in each run. In addition, the efficiency of amplification was calculated for each assay and expression results were corrected for the small differences in efficiency observed between genes (Table 4). All cycle threshold (C_t_) data was scaled relative to a calibrator sample amplified using the same gene.

### Total RNA Isolation

Tissue (50–100 mg) was homogenised in 1 ml of QIAzol Lysis Reagent (Qiagen, Crawley, UK), using a bench-top homogeniser (Polytron PT1600E, Kinematica AG, Littau-Luzem, Switzerland). Total RNA was isolated from homogenised breast tissue using the RNeasy^® ^Tissue Mini Kit (Qiagen, Crawley, UK) according to the manufacturer's instructions. RNA was eluted in 60 μl nuclease-free water and stored at -80°C. In addition to the on-column DNase treatment performed during the RNA extraction procedure, RNA was DNase-treated after extraction using the DNA-free™ DNase Treatment and Removal Reagents (Ambion, Cambridgeshire, UK). RNA concentration and purity was assessed in duplicate samples using a Nanodrop ND-1000 spectrophotometer (Nanodrop Technologies, DE, USA). RNA integrity was assessed using the RNA 6000 Nano LabChip Series II Assay with the 2100 Bioanalyzer System (Agilent Technologies, Palo Alto, CA, USA). Electropherograms and gel-like images were evaluated using the Agilent 2100 Expert software (Version B.02.03) which generated the RNA integrity number (RIN) enabling estimation of RNA integrity. The RIN value describes a graded scale of RNA integrity ranging from 1 (completely degraded total RNA) to 10 (intact total RNA). Based on this tool, total RNA integrity is determined not only by the ratio of the ribosomal bands but by the entire electrophoretic trace of the sample including presence or absence of degradation products [49].

In agreement with recent reports [34,35], there was a significant negative linear relationship between RNA integrity, as determined by analysis of RIN and C_t _values (*P *< 0.05), with increased C_t _values associated with RIN less than 5 (data not shown). Therefore the threshold RIN value for inclusion of RNA samples in analysis was ≥ 7. RNA purity was verified by an average *A*_260_/*A*_280 _ratio of 1.98, ranging from 1.97 to 2.01. *A*_260_/*A*_230 _ratios averaged 1.7, ranging from 1.5 to 1.83.

### First strand cDNA synthesis by reverse transcription

First strand cDNA was synthesised in duplicate reactions for each RNA sample (2 by 1 μg each) using Superscript III reverse transcriptase (Invitrogen Life Technologies, Paisley, UK) and random primers (N_9_; 1 μg; MWG Biotech, AG, Ebersberg, Germany). Negative controls consisting of non-reverse transcribed samples were included in each set of reactions. The reactions were incubated at 25°C for 5 min followed by 50°C for 1 h and finally 72°C for 15 min. Duplicate cDNA reactions were pooled, diluted to 120 μl in nuclease-free water (Invitrogen Life Technologies), aliquotted and stored at -20°C till further use.

### Real-time Quantitative PCR

The expression of the 11 candidate EC genes was analysed by RQ-PCR using TaqMan^® ^Endogenous Control Assays or TaqMan^® ^Gene Expression Assays and the ABI Prism^® ^7000 Sequence Detection System (Applied Biosystems, Foster City, CA). Each gene was tested in triplicate within the same PCR run for the majority of samples, with the remaining samples tested on an additional 96-well plate. TaqMan^® ^Endogenous Control Assay and Gene Expression Assay IDs are listed in Table 4. Samples with standard deviations >0.3 from the mean C_t _of the triplicates were excluded from analysis. *HPRT1*, amplified from pooled normal cDNA, was run on each plate to assess inter-assay variation. cDNA (2 μl) from each tumour sample was added to a PCR reaction mix containing 1× TaqMan^® ^Universal PCR Master Mix, No AmpErase^® ^UNG and 1 μl Endogenous Control Assay or Gene Expression Assay (Applied Biosystems) in a 20 μl reaction volume. Standard cycling conditions were used [95°C for 10 minutes, (95°C for 15 seconds, 60°C for 60 seconds) × 40 cycles]. The inter-assay percent coefficient of variation (%CV) for samples with a mean C_t _of 25.81 ± 0.07 (mean ± s.e.m.) was 0.81% (n = 15).

### PCR amplification efficiencies

Percent PCR amplification efficiencies (E) for each assay were calculated as E = (10^-1/*slope *^- 1) × 100, using the slope of the semi-log regression plot of C_t _versus log input of cDNA (10-fold dilution series of five points) as shown in Table 4. A threshold of 10% above or below 100% efficiency was applied. Amplification efficiencies ranged from 90.9% for *TFRC *to 107.7% for *MRPL19*, indicative of approximate exponential efficiencies for these assays.

### Conversion of cycle threshold (C_t_) to quantity relative to calibrator

The baseline (3–15 cycles) and average threshold cycle (C_t_) were automatically calculated using the ABI Prism SDS Software (version 1.2.3). The C_t _value is defined as the PCR cycle number at which the fluorescence generated from amplification of the target gene within a sample increases to a threshold value of 10 times the standard deviation of the baseline emission and is inversely proportional to the starting amount of target cDNA. C_t _results were converted into quantities relative to normal (Q_Rel_.), and corrected for PCR amplification efficiency (E), using the following formula: Q_Rel_. = E^-ΔCt^, where ΔC_t _= C_t _test sample – C_t _calibrator sample.

### Comparative quantitation of target gene *ESR1 *relative to endogenous control

To calculate the expression of *ESR1*, relative to an EC gene(s), the ΔΔC_t _method was used where ΔΔC_t _= (C_t _target gene, test sample – C_t _endogenous control, test sample) - (C_t _target gene, calibrator sample - C_t _endogenous control, calibrator sample). Fold change in gene expression between groups was calculated as E^-ΔΔCt ^± s.e.m. Where target gene expression was normalised using more than one endogenous control, fold change estimates were calculated using the geometric mean of EC quantities relative to the calibrator sample and the errors were calculated following the rules of error propagation descibed previously [25].

### Analysis of EC stability

Candidate EC gene stability was evaluated using two statistical models for the analysis candidate EC genes, geNorm [[24], Ver. 3.4] and NormFinder [26]. C_t _values were converted into relative quantities considering the PCR amplification efficiencies as detailed above. GeNorm is a Visual Basic application tool for Microsoft^® ^Excel and is freely available by request from the authors [50]. NormFinder is a Microsoft^® ^Excel add-in, also freely available [51]. For NormFinder analysis tissues samples were categorised into benign (n = 6) or malignant groups (n = 21) according to analysis of standard histopathological parameters as described above.

### Statistical analyses

Statistical analyses were performed with Minitab^® ^15 Statistical Software for Windows^® ^(Minitab Ltd., Coventry, UK). *P *values <0.05 were considered statistically significant. The Anderson-Darling normality test was applied. Two-sample *t *tests were used to compare calibrator-scaled EC gene quantities between benign and malignant tissue groups. Equality of variance between scaled EC Q_Rel_. values and effect of EC on the estimate of the error associated with gene expression was analysed using Bartlett's test. Associations between scaled EC and target gene quantities were determined by regression analysis which examined linear, quadratic and cubic relationships. One-way ANOVA was used to compare *ESR1 *expression normalised using different EC genes.

## Abbreviations

RQ-PCR, Real-time quantitative polymerase chain reaction

EC, endogenous control

C_t_, cycle threshold

RIN, RNA integrity number

Q_Rel_., quantity of gene expression relative to normal

NF, geNorm normalisation factor

*V*, geNorm pairwise variation

*M*, geNorm stability measure

T, size or extent of primary tumour

N, spread to regional lymph nodes

M, distant metastasis

UICC, tumour stage according to the International Union Against Cancer TNM classification

ER, oestrogen receptor

PR, progesterone receptor

HER2/*neu*, v-erb-b2 erythroblastic leukaemia viral oncogene

## Authors' contributions

REM conceived, designed and performed the experiments, was responsible for data analyses and for writing the manuscript. NM contributed throughout the experiment and critically reviewed the manuscript. MJK participated throughout as Head of Department, particularly in sample acquisition during surgery and in critically reviewing the manuscript. All authors read and approved the final manuscript.
